# Efficacy and safety of riociguat replacing PDE-5is for patients with pulmonary arterial hypertension: A systematic review and meta-analysis

**DOI:** 10.3389/fphar.2023.1052546

**Published:** 2023-01-26

**Authors:** Yu-Yang Liu, Yi-Yang Qu, Shang Wang, Ci-Jun Luo, Hong-Ling Qiu, Hui-Ting Li, Ping Yuan, Lan Wang, Jin-Ling Li, Rong Jiang, Rui Zhang

**Affiliations:** ^1^ Department of Cardiopulmonary Circulation, Shanghai Pulmonary Hospital, School of Medicine, Tongji University, Shanghai, China; ^2^ Department of Cardiology, Affiliated Hospital of Qingdao University, Qingdao, China

**Keywords:** pulmonary arterial hypertension, riociguat, systematic review, meta, analysis, phosphodiesterase-5 inhibitor

## Abstract

**Introduction:** Pulmonary arterial hypertension (PAH) is a rare and progressive disease. Some patients treated with phosphodiesterase type 5 inhibitors (PDE-5is) fail to reach treatment goals. As a novel soluble guanylate cyclase agonist, riociguat acts on the same pathway as PDE-5is but functions *via* different mechanisms. Whether riociguat is more effective and safer than PDE-5is is ambiguous. We aimed to evaluate the efficacy and safety of switching from PDE-5is to riociguat among these patients.

**Methods:** Original published articles were retrieved from PubMed/Medline, Embase, Web of Science, Open Grey and Google Scholar. Studies that assessed the World Health Organization functional class (WHO-FC), 6-min walking distance (6MWD), pulmonary vascular resistance (PVR), mean pulmonary arterial pressure (mPAP), cardiac index (CI) and N-terminal pro-B-type natriuretic peptide (NT-proBNP) were collected. Adverse events after switching were evaluated.

**Results:** Ten published studies were included. Compared to PDE-5is, riociguat significantly increased the 6MWD by 26.45 m weighted mean difference (WMD) = 26.45 m, 95% confidence intervals (CIs): 9.70–43.2 m, *p* = 0.002) and improved mPAP (WMD = −3.53, 95% CIs: −5.62–1.44 mmHg, *p* = 0.0009), PVR (WMD = −130.24 dyn·s·cm^−5^, 95% CI −187.43–73.05, *p <* 0.0001), CIs (WMD = 0.36 L/min·cm^−2^, 95% CIs: 0.25–0.47, *p <* 0.00001) and WHO-FC (OR = 0.11, 95% CIs: 0.08–0.16, *p* < 0.0001) but not NT-proBNP. In addition, we did not observe the most common side effects during the replacement of riociguat for PDE-5is.

**Conclusions:** Compared to PDE5i, PAH patients benefit more from riociguat in hemodynamics, 6MWD, WHO-FC and biomarkers.

## Introduction

Pulmonary arterial hypertension (PAH) is a life-threatening disease caused by a variety of factors. It results in a progressive increase in pulmonary vascular resistance (PVR), leading to pulmonary vascular remodeling and progressive development of right heart failure, which can lead to death. If untreated, PAH has a very poor prognosis, with a 1-year survival rate of only 69% and a 5-year survival rate of 38% (Aaron and Roham, 2013; Pulido et al., 2016). The pathogenesis of PAH involves complex mechanisms, such as vascular dysfunction and an imbalance in the activities of the endothelin pathway, prostacyclin pathway, and nitric oxide (NO) pathway (Ataya et al., 2016). There are three major classes of PAH-targeted drugs approved for PAH: endothelin receptor antagonists, prostacyclin analogs, and drugs targeting the NO-soluble guanylate cyclase (sGC)-cyclic guanosine monophosphate (cGMP) pathway. Three drugs targeting the NO-sGC-cGMP pathway are approved for the treatment of PAH: two phosphodiesterase-5 inhibitors (PDE-5is), sildenafil and tadalafil, and the sGC stimulator riociguat (Aaron and Roham, 2013; Ataya et al., 2016; Pulido et al., 2016; Barnes et al., 2019).

Phosphodiesterase type 5 inhibitors (PDE-5is) relax pulmonary smooth muscle by releasing the bioactive substance NO and were first used in the treatment of PAH in 2003 (Michelakis et al., 2003). However, the effects of PDE-5is are dependent on the level of NO. With the progression of PAH, the concentration of NO gradually decreases, and the effects of PDE-5is are reduced. The clinical deterioration of a proportion of PAH patients occurs due to an insufficient response to or intolerance of PDE-5is (Aleevskaya et al., 2020), a considerable drawback of PDE-5is for PAH patients who require long-term treatments (Barnes et al., 2019).

As another NO pathway PAH-targeted drug, riociguat is a novel soluble guanylate cyclase (sGC) agonist for PAH treatment that was developed in recent years (Ghofrani et al., 2013a). Riociguat acts on the same pathway as PDE-5is, but the mechanism is different. Riociguat increases intracellular cyclic guanosine monophosphate (cGMP) levels by directly stimulating the sGC enzyme, independent of NO levels. Therefore, riociguat can have effects on PAH patients whether the level of NO is sufficient in the early stage or decompensated in the late stage (Hoeper et al., 2021). In the PATENT and CHEST studies, riociguat significantly reduced PVR and improved the 6-min walking distance (6MWD), WHO functional class (WHO-FC) and subjective symptom scores among PAH and chronic thromboembolic pulmonary hypertension (CTEPH) patients (Ghofrani et al., 2013a; Ghofrani et al., 2013b). Interestingly, in the recent RESPITE study, selected patients with PAH benefitted from switching from PDE-5is to riociguat when patients did not reach treatment goals with PDE-5i therapy (Hoeper et al., 2017). According to the 2022 ESC/ERS guidelines for pulmonary hypertension, for patients with IPAH/HPAH/DPAH who present with an intermediate-low risk of death while receiving ERA/PDE-5i therapy, switching from PDE-5is to riociguat may be considered (IIb) (Humbert et al., 2022). However, there is limited medical evidence at present.

Therefore, the purpose of this meta-analysis was to comprehensively evaluate the efficacy and safety of replacing PDE-5is with riociguat to provide evidence for PAH patient therapy.

## Methods

We followed the protocol (PRlOSPERO: CRD 42022304966) and the criteria in the PRISMA statement (Supplementary Table S1) for this meta-analysis.

### Literature search

A comprehensive literature search was performed in electronic databases, including PubMed/Medline, Embase, Web of Science, Open Grey and Google Scholar, from January 1, 2013, to December 31, 2021. The language was limited to English. The search keywords were “pulmonary arterial hypertension”, “Adempas”, “riociguat”, “BAY 63-2521”, “tadalafil”, “sildenafil” and “phosphodiesterase-5 inhibitors”. The refined search formula was ((pulmonary hypertension [Title/Abstract]) AND (therapy [Title/Abstract])) AND ((tadalafil [Title/Abstract]) OR (sildenafil [Title/Abstract]) OR (phosphodiesterase-5 inhibitors [Title/Abstract])) AND ((Adempas [Title/Abstract]) OR (Riociguat[Title/Abstract])). References of all relevant studies were further reviewed by two reviewers to ensure that all relevant studies were identified.

### Inclusion and exclusion criteria

Articles from all databases were included if they met the following criteria:

i: PAH patients with WHO-FC II or greater, mean pulmonary arterial pressure (mPAP) ≥ 25 mm Hg and pulmonary arterial wedge pressure ≤ 15 mm Hg;
ii: All patients received stable doses of PDE-5is (tadalafil, sildenafil or others) as monotherapy or in combination with other drug classes;
iii: Patients with side effects caused by PDE-5is or patients who responded inadequately to PDE-5i therapy;
iv: The mean (Mean) and standard deviation (SD) were directly or indirectly obtainable from the article; and
v: The studies were non-randomized controlled trials (NRCTs) that were controlled before-after or cohort studies.

The exclusion criteria were as follows:

i: Patients previously treated with riociguat;
ii: Patients with clinically significant restrictive or obstructive pulmonary parenchymal disease and left heart failure;
iii: Patients who had a low-risk status or had a better treatment effect with PDE-5is;
iv: Patients who received riociguat as an add-on therapy without PDE-5i replacement; and v: Cohort studies with a small sample size, large bias or a Newcastle‒Ottawa Scale (NOS) score ≤ 7 points.

### Data extraction

The data were independently extracted by two investigators directly or indirectly using Engauge Digitizer software (Markmitch), version 4.1, to obtain required data from tables and images. The extracted content mainly included the basic characteristics of the included studies, including first author, publication year, study design type, sample size, sex, age, classification of PAH, WHO-FC, baseline status, tadalafil or sildenafil dose, riociguat dose, washout period, drug adjustment period, etc. (Table 1).

**TABLE 1:** Basic characteristics of the included literature.

Author year	Range/Study design/Sample size	ITT/Baseline description/Discontinued	PDE-5i pretreatment		Riociguat dose	Washout period	Adjustment period	Endpoint (n)
	Sex/Age		Sildenafil	Tadalafil				
	PH classification/WHO-FC							
Hoeper M. M. 2021	22 countries; RCT; 111	Yes/REF/7	≥60 mg per day	20–40 mg per day	2.5 mg/d	Sildenafil: 24 h, Tadalafil: 48 h	24 w	45
	M29, F82; 18–75							
	PAH, POPH, CTD-PAH; FC III							
Kuroda K. 2019	Japan; PCS; 7	Yes/Yes/2	40 mg	40 mg	5.7 ± 2.2 mg/d	Sildenafil: 12 h; Tadalafil: 24 h	265 d	7
	M3, F4; 50 ± 20							
	PAH; FC II, III							
Hoeper M. M. 2017	Europe and United States; RCS; 61	No/REF/10	20–80 mg, tid	40 mg/d	2.5 mg, tid	Sildenafil: 1–3 d; Tadalafil: 72 h	24 w	16
	M16, F45; 18–75							
	PAH; FC III							
Gall H. 2018	International; RCS; 98	Yes/Yes/0	Unknown		3.37 mg/d	Sildenafil: −24 to 13 d; Tadalafill: −1–5 d	5 m	125
	M49, F76; 64 ± 16							
	PAH, CTEPH; FC III							
Taran I. N. 2018	Russia; PCS; 8	Yes/REF/0	20–80 mg tid	None	7.5 mg/d	24 h	12 w	8
	M2, F6; 47.0 (39.5–50.2)							
	IPAH; FC II, III							
Darocha S. 2018	Poland; RCS; 28	Yes/REF/0	25 mg, tid	None	2.5 mg, tid	Unknown	22.3 m	28
	M10, F18; 65 ± 15							
	CTEPH; FC I, II, III							
Aleevskaya A. 2020	Russia; PCS; 12	Yes/REF/0	60–80 mg, tid	None	Unknown	Unknown	13.5 m	12
	--; 39.2 ± 10.7							
	IPAH; FC II, III							
Davey R. A. 2017	United States; RCS; 8	Yes/REF/0	Unknown		2.4 ± 0.1 mg, tid	Unknown	172 w	8
	M2, F10; 58							
	Japan; PAH, CTEPH; FC 2.8							
Weir N. 2017	United States; RCS; 31	Yes/REF/9	Unknown		Unknown	Unknown	Unknown	22
	--; --							
	PAH; --							
Yamamoto 2017	PCS; 8	Yes/REF/0	Unknown		6.5 ± 1.4 mg/d	26.5 ± 10 h	6–12 m	8
	M1, F7; 72.8 ± 3.1							
	CTEPH; FC II, III							

Note: CTD-PAH, connective tissue disease-associated pulmonary arterial hypertension; CTEPH, chronic thromboembolic pulmonary hypertension; F, female; IPAH, idiopathic pulmonary arterial hypertension; ITT, intention-to-treat analysis; M, male; PAH, pulmonary artery hypertension; PCS, prospective cohort studies; POPH, portopulmonary hypertension; RCS, retrospective cohort studies; RCT, randomized controlled trial; REF, referred (but no description); United States, The United States of America; WHO-FC, the world health organization functional class.

### Outcomes

The outcomes included changes in 6MWD, WHO-FC, hemodynamic variables and N-terminal pro-B-type natriuretic peptide (NT-proBNP) from baseline to the end of the study. The hemodynamic variables included mPAP, PVR, and cardiac index (CI). The adverse events included but were not limited to headache, peripheral edema, dyspepsia, dizziness, diarrhea, nausea, syncope, vomiting, dyspnea, nasopharyngitis, anemia, gastroesophageal reflux disease, palpitations, hypotension, chest pain, coughing, tachycardia, nasal congestion, fever, chest discomfort, gastritis, fatigue, and flushing.

### Quality assessments

Two researchers independently completed the quality evaluation. If there was any disagreement, a third researcher was consulted.

The literature quality of the cohort study was assessed using the NOS (Wells et al., 2000), which consists of the following aspects:

1) the representativeness of research subjects and the outcome indicators at the beginning of the study;
2) the comparability of exposed and non-exposed groups; and
3) the criteria of the outcome assessment, the follow-up period, and the completeness of the follow-up. The NOS scores ranged from 0–9, with a score ≥ 7 considered high quality.

The risk of bias for the randomized controlled trial (RCT) was evaluated using version 2 of the Cochrane tool for assessing risk of bias in randomized trials (RoB2) (Sterne et al., 2019), including selection bias, performance bias, detection bias, attrition bias, reporting bias and other bias.

### Statistical analysis

In this meta-analysis, the outcomes were analyzed using Review Manager software, version 5.4.1, and STATA software, version 12. For continuous data, the mean difference or standardized mean difference and 95% confidence intervals (*CI*s) were analyzed. The *I*-squared (*I* ^2^) test was used to analyze the heterogeneity between different studies. Significant heterogeneity was considered when *I* ^2^ was > 50%, and a random effects model was used to analyze the pooled outcomes. Conversely, a fixed effects model was applied when no significant heterogeneity was observed. A pooled effects model with weighted mean difference (WMD) and 95% *CI*s was used to assess the prognosis with riociguat. The stability of the meta-analysis results was assessed by sensitivity analysis. Report bias was checked by funnel plots. Since WHO-FC was ordinal data, it was no longer appropriate to use the standardized mean difference (SMD) of the continuous outcome effect as the effect value, so multicategory logistic regression analysis was applied. In logistic regression, the effect value is the odds ratio (OR) for measuring the effect of exposure factors. If the OR = 1, there is no correlation between the exposure and outcome; if the OR > 1, the exposure is a contributing factor to the occurrence of the outcome; and if the OR < 1, the exposure is an outcome inhibitory factor. In this study, the “exposure” referred to the replacement of PDE-5is with riociguat, and the “outcome” was the improvement in WHO-FC. A *p*-value less than 0.05 was considered statistically significant.

## Results

### Description of all included studies

**FIGURE 1:**
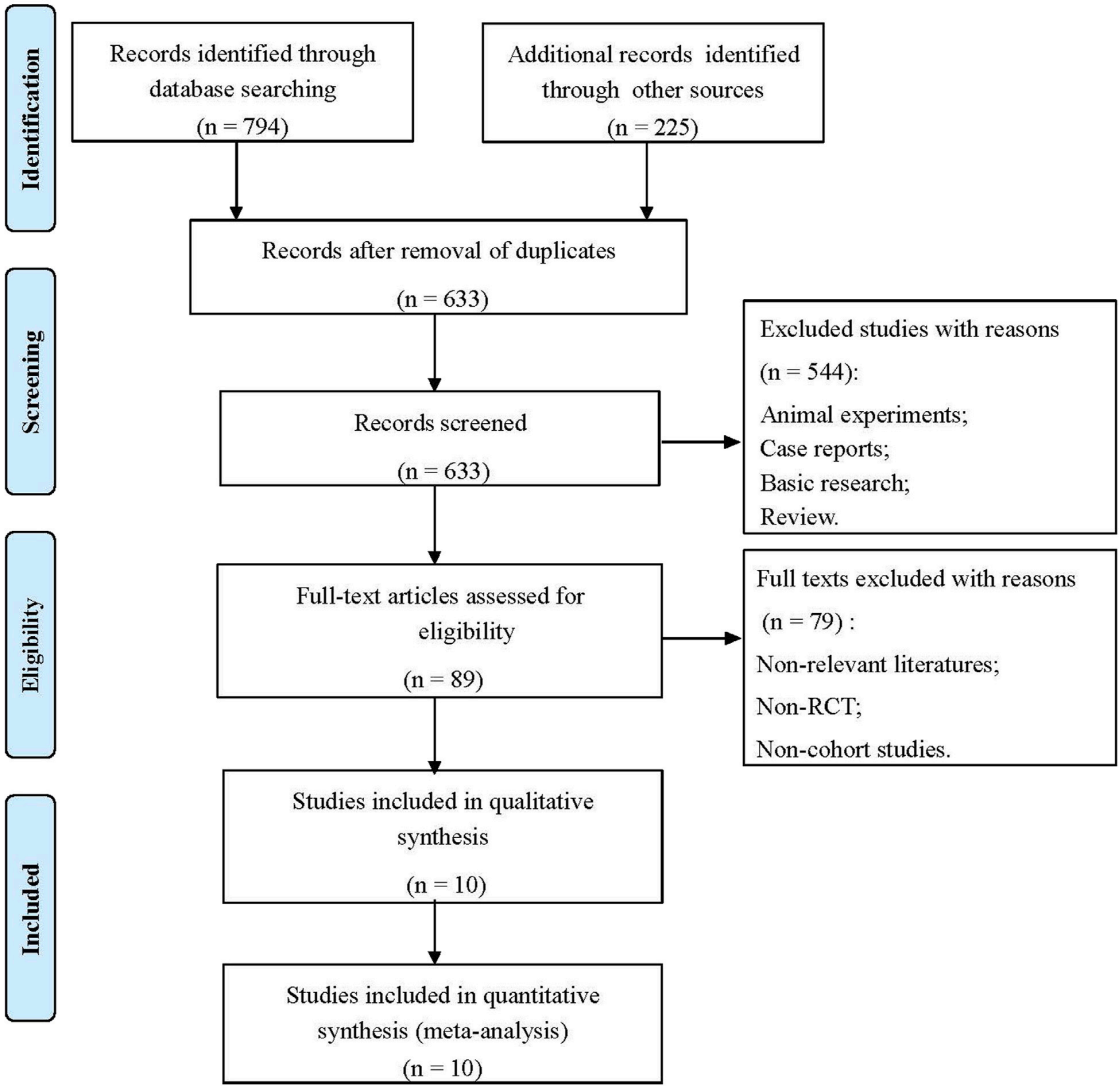
Flowchart of the study selection process in this meta-analysis.

The flowchart of the literature screening process is shown in Figure 1. Through a double search, a total of 1,019 studies were retrieved after searching the electronic databases. A total of 386 studies were removed due to duplication, and 544 studies were removed for being case reports, basic research, animal experiments, reviews, etc. After reading the full texts, 79 articles were excluded because they were non-relevant studies, non-RCTs or non-cohort studies. Finally, 10 studies met the inclusion criteria and were selected, including one RCT (Hoeper et al., 2021), five prospective cohort studies (Hoeper et al., 2017; Yamamoto et al., 2017; Taran et al., 2018; Aleevskaya et al., 2020; Kuroda et al., 2020), and four retrospective cohort studies (Davey et al., 2017; Weir et al., 2017; Darocha et al., 2018; Gall et al., 2018). A total of 376 patients were involved. In addition, the results of the risk of bias assessment are shown in Supplementary Figure S1; Supplementary Table S2.

#### 6MWD

**FIGURE 2:**
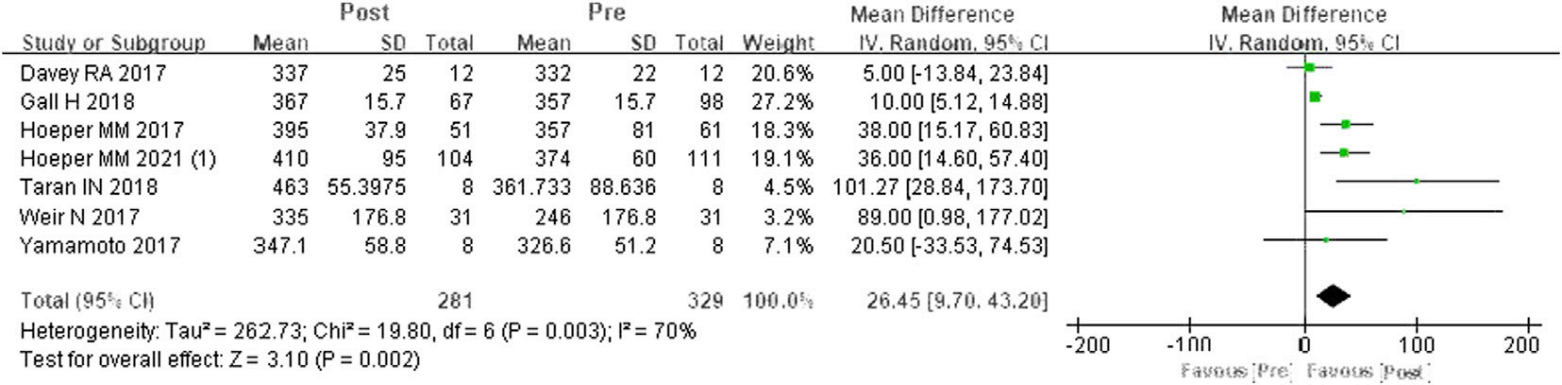
Changes in 6MWD after PDE-5is were replaced by riociguat. 6MWD: 6-min walking distance; PDE-5is: phosphodiesterase-5 inhibitors.

A total of seven studies were included (Davey et al., 2017; Hoeper et al., 2017; Weir et al., 2017; Yamamoto et al., 2017; Gall et al., 2018; Taran et al., 2018; Hoeper et al., 2021). According to the heterogeneity test (*I* ^*2*^ = 69.7%, *p* = 0.003), there was statistical heterogeneity among the studies, so a random effects model was applied. The meta-analysis results showed that, compared to PDE-5is, riociguat could obviously improve 6MWD by an average of 26.45 m, and the difference was statistically significant (WMD = 26.45 m, 95% CIs: 9.70–43.2, *p* = 0.002) (Figure 2).

#### mPAP

**FIGURE 3:**
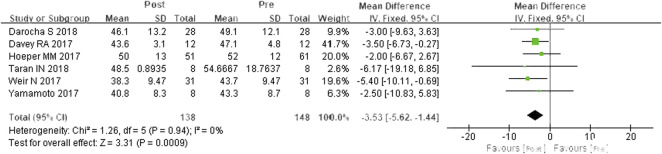
Changes in mPAP after riociguat replaced PDE-5i therapy. mPAP: mean pulmonary arterial pressure; PDE-5i: phosphodiesterase-5 inhibitor.

A total of six studies were included (Davey et al., 2017; Hoeper et al., 2017; Weir et al., 2017; Yamamoto et al., 2017; Darocha et al., 2018; Taran et al., 2018). According to the heterogeneity test (*I* ^*2*^ = 0%, *p* = 0.94), there was no statistical heterogeneity among the studies, so a fixed effects model was applied. In contrast to PDE-5is, riociguat decreased mPAP by 3.53 mmHg, and the differences were statistically significant (WMD = 3.53 mm Hg, 95% CIs: −5.62-1.44, *p* = 0.0009) (Figure 3).

#### PVR

**FIGURE 4:**
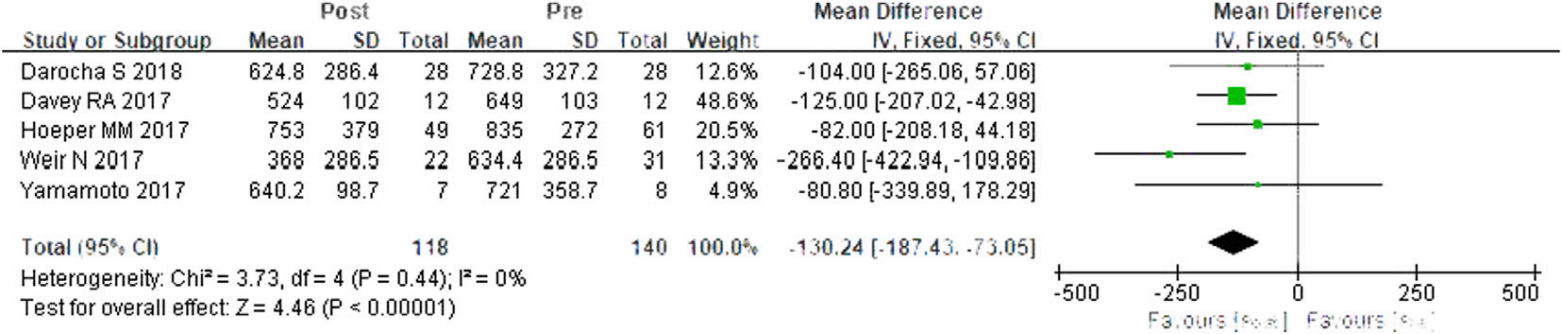
Changes in PVR after riociguat replaced PDE-5i therapy. PVR: pulmonary vascular resistance; PDE-5i: phosphodiesterase-5 inhibitor.

A total of five studies were included (Davey et al., 2017; Hoeper et al., 2017; Weir et al., 2017; Yamamoto et al., 2017; Darocha et al., 2018). According to the heterogeneity test (*I* ^*2*^ = 0%, *p* = 0.444), there was little statistical heterogeneity among the studies, so a fixed effects model was applied. The meta-analysis results showed that PVR was decreased by 130.24 dyn·s·cm^−5^ after switching from PDE-5is to riociguat, and the difference was statistically significant (WMD = 130.24 dyn·s·cm^−5^, 95% CIs: −187.43–73.05, *p <* 0.0001) (Figure 4).

#### CI

**FIGURE 5:**
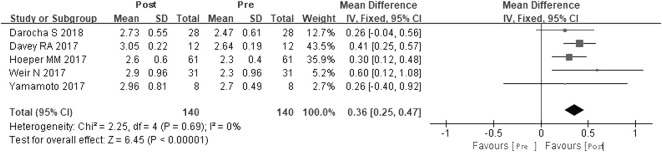
Changes in CI after riociguat replaced PDE-5i therapy. CI: cardiac index; PDE-5i: phosphodiesterase-5 inhibitor.

A total of five studies were included (Davey et al., 2017; Hoeper et al., 2017; Weir et al., 2017; Yamamoto et al., 2017; Darocha et al., 2018). According to the heterogeneity test (*I* ^*2*^ = 0, *p* = 0.69), the statistical heterogeneity among the studies was small, so a fixed effects model was applied. The results showed that riociguat could increase CI by 0.36 L/min·cm^−2^, and the difference was statistically significant (WMD = 0.36 L/min·cm^−2^, 95% CIs: 0.25–0.47, *p <* 0.00001) (Figure 5).

#### WHO-FC

A total of seven studies were included (Hoeper et al., 2017; Yamamoto et al., 2017; Darocha et al., 2018; Gall et al., 2018; Taran et al., 2018; Aleevskaya et al., 2020; Hoeper et al., 2021). Using fixed effects model analysis, *p* < 0.0001, the result was statistically significant (OR = 0.11, 95% CIs: 0.08–0.16). The results showed that WHO-FC decreased significantly after PDE-5is were replaced by riociguat.

#### NT-proBNP

A total of five studies were included (Davey et al., 2017; Hoeper et al., 2017; Gall et al., 2018; Kuroda et al., 2020; Hoeper et al., 2021). According to the heterogeneity test (*I* ^*2*^ = 73%, *p* = 0.005), there was statistical heterogeneity among the studies, so a random effects model was applied. The meta-analysis results showed that NT-proBNP decreased after switching to riociguat, but the difference was not statistically significant (SMD = −0.25, 95% CIs: −0.64–0.14, *p* = 0.20) (Supplementary Figure S2).

#### Safety

In the REPLACE study (Hoeper et al., 2021), overall adverse events were reported in similar proportions of patients in the riociguat group and the PDE-5i group. The most frequently occurring adverse events were hypotension (15.14%), headache (14.13%), and dyspepsia (10.9%) in the riociguat group and headache (8.7%), coughing (7.6%), and upper respiratory tract infection (7.6%) in the PDE-5i group. Serious adverse events (SAEs) were less frequent in the riociguat group (8.7%) than in the PDE-5i group (19.17%), including hypotension in the riociguat group and pneumonia and PAH in the PDE-5i group. Gall et al. (2018) reported that the most common adverse events (AEs) were dizziness (11.9%), dyspepsia (10.8%), and headache (6.5%) among PAH and CTEPH patients who switched to riociguat from another PAH-targeted therapy. SAEs occurred among six patients, two of which were study drug-related events, while one patient experienced palpitations, a viral infection, and cardiac catheterization, and another patient experienced right ventricular failure. In another study (Kuroda et al., 2020), systolic blood pressure (SBP) decreased after switching to riociguat, and SBP increased from 97 ± 13–106 ± 18 mm Hg during follow-up. The most common side effects were not observed after the transition from a PDE-5i to riociguat in this study.

### Publication bias and sensitivity analysis

The analysis funnel plots of 6MWD, mPAP, PVR and CI showed that the data were symmetrical relative to the midline in each group, and the publication bias was small (Supplementary Figures S3A–D); the analysis funnel plots of NT-proBNP showed poor data symmetry and high publication bias (Supplementary Figure S3E). In the analysis of 6MWD, mPAP, PVR, and CI, the combined results of the included studies changed little, indicating that the study quality of this group was credible, and the heterogeneity was low (Supplementary Figure S3F-I); in the analysis of NT-proBNP, the combined results of the included studies changed greatly after excluding some single studies. This outcome indicated that the study quality of this group was poor and that heterogeneity was high. This result might be caused by the limited sample size of the selected studies (Supplementary Figure S3J).

## Discussion

Our study showed that riociguat, as an sGC agonist, has good clinical efficacy for PAH patients. The replacement of PDE-5is with riociguat could improve 6MWD, mPAP, PVR, CI, and WHO-FC in PAH patients.

Recently, due to the rapid development and clinical application of new drugs, the median survival period of patients with PAH has significantly improved. A 2012 NIH-funded registry study found that the estimated average three-, five-, and seven-year survival rates for PAH patients have improved substantially to approximately 92%, 74%, and 65%, respectively (Benza et al., 2012). Therefore, evaluation of the efficacy and safety of new drugs is of great importance for the clinical selection of treatment options and improvement of patient outcomes.

For patients without a sustained response to PDE-5is, there is a biological rationale for switching to sGC stimulators. In healthy subjects, cGMP production in pulmonary vascular smooth muscle cells (PVSMCs) arises from the activation of sGC by NO released from neighboring endothelial cells (Archer et al., 1998). PDE5 mediates degradation of cGMP in the pulmonary circulation. Inhibiting PDE5 activity increases intracellular cGMP levels in situations of impaired NO signaling, resulting in vasodilation and inhibition of PVSMC proliferation (Ghofrani et al., 2006). However, in patients with PAH, evidence suggests that NO bioavailability is reduced. During disease progression, there is a decline in endothelial function, resulting in NO depletion and low intracellular cGMP concentrations, which can render PDE-5is ineffective (Giaid and Saleh, 1995; Archer et al., 1998). Riociguat directly stimulates sGC, increasing intracellular cGMP in the presence or absence of NO (Stasch et al., 2011). Based on this mode of action, riociguat could benefit patients with an insufficient response to PDE-5is.

Both the sGC agonist riociguat and PDE-5is act through the NO pathway. The effect of PDE-5is is dependent on the level of NO. Although there is a certain effect initially, with the progression of the disease, the level of NO decreases, which could lead to a non-ideal improvement of clinical symptoms and long-term quality of life of patients. Riociguat has a dual mechanism of action to relax the pulmonary artery endothelium by stabilizing NO-sGC and increasing the sensitivity of sGC to endogenous NO. Riociguat directly stimulates sGC through different binding sites, activating the NO-sGC-cGMP pathway and increasing cGMP production, independent of the level of NO. Our study analyzed the effect of replacing PDE-5is with riociguat in patients with PAH and found that, compared with PDE-5is, riociguat has obvious benefits, and the results are consistent with previously published research data.

PATENT-1, a double-blind, multicenter study (Ghofrani et al., 2013b) of 443 subjects with PAH, showed a significant improvement in 6MWD in the riociguat group compared with placebo (76%, n = 193 vs. 59%, n = 74), and there was a significant improvement in 6MWD from the second week of treatment. At 12 weeks of treatment, the riociguat group had a mean increase of 36 m in 6MWD (95% CI: 20–52 m, *p* < 0.0001). In the PATENT-1 study, compared with the placebo group, patients with PAH in the riociguat group had a significant increase in cardiac output of 0.9 L/min (*p* < 0.001) and a significant decrease in mPAP by 4 mm Hg (*p* < 0.001). Patients in the riociguat group also had significant improvements in WHO-FC and PVR compared with the placebo group. The difference in NT-proBNP was 432 pg/mL lower than that in the placebo group (Ghofrani et al., 2013b). Similar results were further validated in the CHEST study of CTEPH patients in a different population (Ghofrani et al., 2013a).

The REPLACE study (Hoeper et al., 2021) published in 2021 was a prospective, randomized, controlled, international, multicenter, two-arm, open-label study that compared benefits among patients with PAH who did not meet targets using stable doses of PDE-5is (with or without ERA) to those in a group among whom riociguat replaced PDE-5is and in a PDE-5i-maintained group. After 24 weeks of treatment, the rate of satisfactory response to the primary composite endpoint in the riociguat group (41%) was twice as high as that in the PDE-5i group (20%), with an OR = 2.78. The secondary endpoints of 6MWD, WHO-FC, NT-proBNP and other indicators were significantly improved in the riociguat group. In our study, we found that PAH patients benefited from PDE-5is compared to riociguat, including for mPAP, PVR, CI, 6MWD, and WHO-FC. Therefore, for those PAH patients who switched to riociguat with inadequate response to phosphodiesterase-5 inhibitors, this treatment strategy was beneficial for their prognosis. However, this also needs to be confirmed by subsequent multicenter large-sample prospective studies. In terms of safety, the proportion of AEs was similar between the two groups. There was no significant difference between the two groups in the safety of replacing PDE-5is with riociguat in terms of the incidence of AEs, but the incidence of SAEs was lower than that in the PDE-5i group. The overall safety profile was consistent with previously published safety data on riociguat.

Due to the rare incidence of PAH, it is difficult to systematically evaluate when and how to switch patients with PAH who have failed PDE-5i therapy to riociguat. Therefore, this study could assist clinicians in determining which patients might benefit from transitioning to riociguat, i.e., PAH patients who have WHO-FC III, dyspnea, worsening 6MWD results, or worsening hemodynamic parameters.

### Study limitations

Our study has some limitations. First, among the 10 studies we included, there was only one randomized controlled trial. Therefore, no subgroup analysis was performed in our study, which to some extent increased the risk of bias in the design and implementation of the experiment, and this was not conducive to improving the statistical validity of our study. Second, a small sample size and a large number of retrospective studies limited the representativeness of our study overall and reduced the persuasiveness of the research results to some extent. Third, some of the studies included were only intended to explore the safety of switching from PDE-5is to riociguat, which also affected the value of the study results. Fourth, there were no adverse event data in many studies before drug switching. Finally, our meta-analysis could not completely exclude selection bias. Riociguat directly stimulates sGC, increasing intracellular cGMP in the presence or absence of NO. Based on this mode of action, riociguat could benefit patients with an insufficient response to PDE-5is. However, this hypothesis has not yet been proven. Therefore, additional data from larger RCTs are needed to further establish the efficacy of replacing PDE-5is with riociguat.

## Conclusion

The results of this meta-analysis suggested that switching from PDE-5is to riociguat could efficiently improve the hemodynamic parameters and 6MWD of patients with PAH. Therefore, riociguat might be a viable treatment escalation option for these patients.

## Data Availability

The raw data supporting the conclusion of this article will be made available by the authors, without undue reservation.
