# Corrigendum: Efficacy and safety of riociguat replacing PDE-5is for patients with pulmonary arterial hypertension: A systematic review and meta-analysis

**DOI:** 10.3389/fphar.2023.1155631

**Published:** 2023-02-20

**Authors:** Yu-Yang Liu, Yi-Yang Qu, Shang Wang, Ci-Jun Luo, Hong-Ling Qiu, Hui-Ting Li, Ping Yuan, Lan Wang, Jin-Ling Li, Rong Jiang, Rui Zhang

**Affiliations:** ^1^ Department of Cardiopulmonary Circulation, Shanghai Pulmonary Hospital, School of Medicine, Tongji University, Shanghai, China; ^2^ Department of Cardiology, Affiliated Hospital of Qingdao University, Qingdao, China

**Keywords:** pulmonary arterial hypertension, riociguat, systematic review, meta, analysis, phosphodiesterase-5 inhibitor

In the published article, there was an error.

A correction has been made to the conclusions of the abstract on page one. This sentence previously stated:

“PAH patients benefit from PDE-5is compared to riociguat, including in hemodynamic parameters, 6MWD, WHO-FC and biomarkers.”

The corrected sentence appears below:

“Compared to PDE5i, PAH patients benefit more from riociguat in hemodynamics, 6MWD, WHO-FC and biomarkers.”

The authors apologize for this error and state that this does not change the scientific conclusions of the article in any way. The original article has been updated.

