# Supplementary figures and images for: Efficacy and safety of riociguat replacing PDE-5is for patients with pulmonary arterial hypertension: A systematic review and meta-analysis
Source: Front Pharmacol. 2023 Jan 26;14:1052546. doi: 10.3389/fphar.2023.1052546 (PMC9909097; doi:10.3389/fphar.2023.1052546)

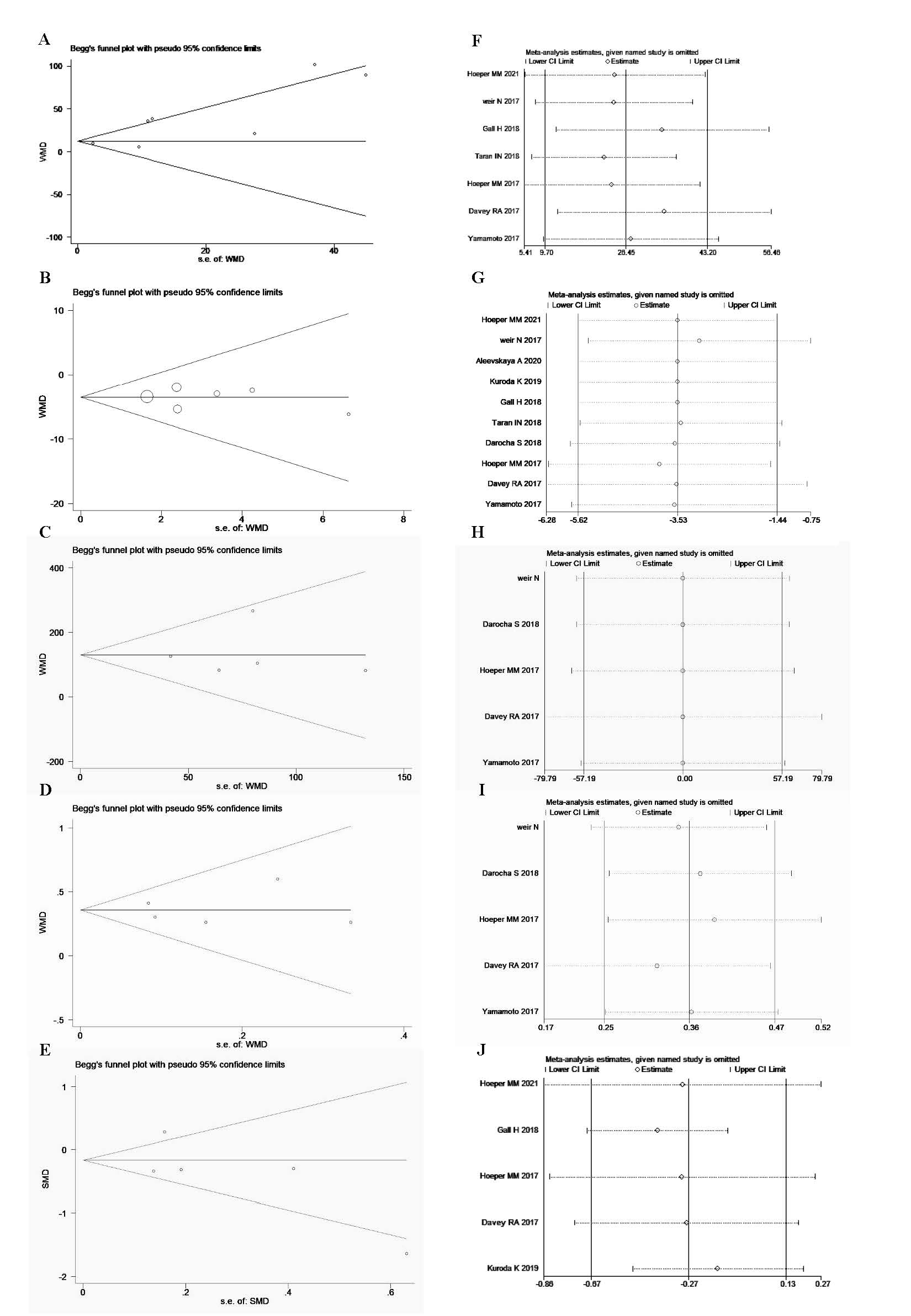

Supplement: Supplementary file 1 [file Image3.JPEG]

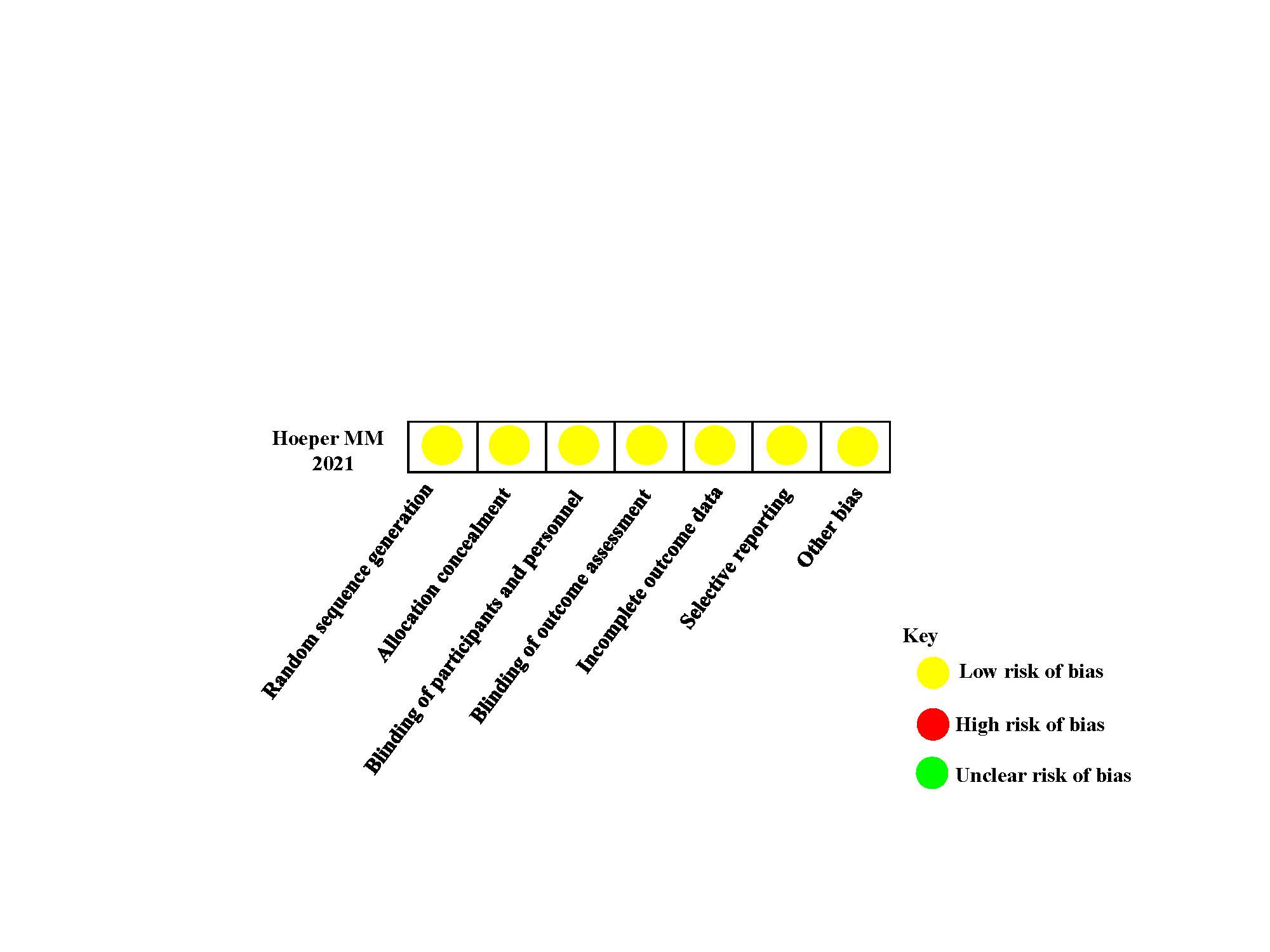

Supplement: Supplementary file 2 [file Image1.JPEG]

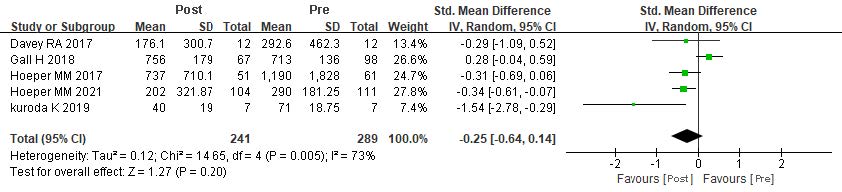

Supplement: Supplementary file 3 [file Image2.JPEG]
