# Supplementary material for: Efficacy and safety of riociguat replacing PDE-5is for patients with pulmonary arterial hypertension: A systematic review and meta-analysis
Source: Front Pharmacol. 2023 Jan 26;14:1052546. doi: 10.3389/fphar.2023.1052546 (PMC9909097; doi:10.3389/fphar.2023.1052546)
Supplement: Supplementary file 5 [file Table2.docx]

**Supplementary Table 2 Newcastle-Ottawa Scale for cohort studies**

| **Study** | | Weir N  2017 | Aleevskaya A  2020 | Kuroda K  2019 | Gall H  2018 | Taran IN  2018 | Darocha S  2018 | Heoper MM  2017 | Davey RA  2017 | Yamamoto  2017 |
| --- | --- | --- | --- | --- | --- | --- | --- | --- | --- | --- |
| **Selection** | Representativeness of the exposed cohort | ☆ | ☆ | ☆ | ☆ | ☆ | ☆ | ☆ | ☆ | ☆ |
|  | Selection of the non-exposed cohort |  |  |  |  | ☆ |  |  |  |  |
|  | Ascertainment of exposure | ☆ | ☆ | ☆ | ☆ | ☆ | ☆ | ☆ | ☆ | ☆ |
|  | Demonstration that outcome of interest was not present at start of study | ☆ | ☆ | ☆ | ☆ | ☆ | ☆ | ☆ | ☆ | ☆ |
| **Compar-ability** | Comparability of cohorts on the basis of the design or analysis |  |  |  |  | ☆☆ | ☆ | ☆ |  |  |
| **Exposure** | Assessment of outcome | ☆ | ☆ | ☆ | ☆ | ☆ | ☆ | ☆ | ☆ | ☆ |
|  | Was follow-up long enough for outcomes to occur | ☆ | ☆ |  | ☆ |  | ☆ | ☆ | ☆ | ☆ |
|  | Adequacy of follow up of cohorts |  |  |  | ☆ | ☆ | ☆ | ☆ |  | ☆ |
| **Score** | | 6 | 6 | 5 | 6 | 8 | 7 | 7 | 5 | 6 |

**NEWCASTLE - OTTAWA QUALITY ASSESSMENT SCALE COHORT STUDIES**

Note: A study can be awarded a maximum of one star for each numbered item within the Selection and Outcome categories. A maximum of two stars can be given for Comparability

**Selection**

1) Representativeness of the exposed cohort

a) truly representative of the average _______________ (describe) in the community **☆**

b) somewhat representative of the average ______________ in the community **☆**

c) selected group of users eg nurses, volunteers

d) no description of the derivation of the cohort

2) Selection of the non-exposed cohort

a) drawn from the same community as the exposed cohort **☆**

b) drawn from a different source

c) no description of the derivation of the non-exposed cohort

3) Ascertainment of exposure

a) secure record (eg surgical records) **☆**

b) structured interview **☆**

c) written self-report

d) no description

4) Demonstration that outcome of interest was not present at start of study

a) yes **☆**

b) no

**Comparability**

1) Comparability of cohorts on the basis of the design or analysis

a) study controls for _____________ (select the most important factor) **☆**

b) study controls for any additional factor **☆** (This criteria could be modified to indicate specific control for a second important factor.)

**Outcome**

1) Assessment of outcome

a) independent blind assessment **☆**

b) record linkage **☆**

c) self report

d) no description

2) Was follow-up long enough for outcomes to occur

a) yes (select an adequate follow up period for outcome of interest) **☆**

b) no

3) Adequacy of follow up of cohorts

a) complete follow up - all subjects accounted for **☆**

b) subjects lost to follow up unlikely to introduce bias - small number lost - > ____ % (select an adequate %) follow up, or description provided of those lost) **☆**

c) follow up rate < ____% (select an adequate %) and no description of those lost

d) no statement
